# A complete durable response of vaginal clear cell carcinoma with pembrolizumab: A case report

**DOI:** 10.1016/j.gore.2023.101160

**Published:** 2023-03-11

**Authors:** Hector S. Porragas-Paseiro, Saketh Guntupalli, Jessie Xiong, Ashley Greenwood

**Affiliations:** a University of Colorado School of Medicine, Department of Obstetrics and Gynecology, Division of Gynecologic Oncology, United States; b University of Colorado School of Medicine, Department of Pathology, United States

**Keywords:** Diethylstilbestrol, Clear cell carcinoma, Pembrolizumab

## Abstract

Primary clear cell adenocarcinoma of the vagina represents a rare form of cancer historically correlated with in-utero diethylstibestrol (DES) exposure. Mainstay treatment modalities include surgery, radiation, and chemotherapy. There has been a growing interest in immunotherapy in the field of oncology. KEYNOTE 826 demonstrated that patients with persistent, recurrent, or metastatic cervical cancer including patients who had adenocarcinoma showed improved progression and overall survival by the addition Pembrolizumab to chemotherapy plus or minus bevacizumab. To date, there are no documented cases using pembrolizumab as adjuvant treatment for active or recurrent vaginal clear cell adenocarcinoma. We present a case of a young patient with recurrent vaginal clear cell carcinoma who showed a complete and durable response to Pembrolizumab.

## Highlights

• Due to the rarity of primary vaginal carcinoma, treatment practices are extrapolated from cervical or vulvar data.
• In-utero diethylstilbestrol exposure is correlated with vaginal cell carcinoma.
• Pembrolizumab can provide a durable response to vaginal clear cell carcinoma.

## 1 Introduction

Diethylstilbestrol (DES) is a synthetic form of estrogen that was prescribed from 1938 to 1971 to help prevent pregnancy complications such as miscarriage and premature labor (Ervin, 1976). In the 1960s a cluster of young women 15–22 years old were identified to have clear cell or epithelioid adenocarcinoma of the vagina. This was a very unusual finding as vaginal cancers are rare, commonly squamous histology, and mostly affect women greater than 50 years of age (Adams and Cuello, 2018). It was identified that there was a strong correlation between maternal in utero DES exposure and clear cell adenocarcinoma of the female lower genital tract (Herbst et al., 1971). While the number of women exposed to DES in utero is unknown, it is estimated that 2–4 million pregnant women were treated with DES or similar synthetic estrogens during their pregnancy (Huo et al., 2017). Due to the adverse outcomes, DES was pulled from the US market in 1971 but was continued in other countries up to the 1980s (Herbst et al., 1971).

**Fig. 1:**
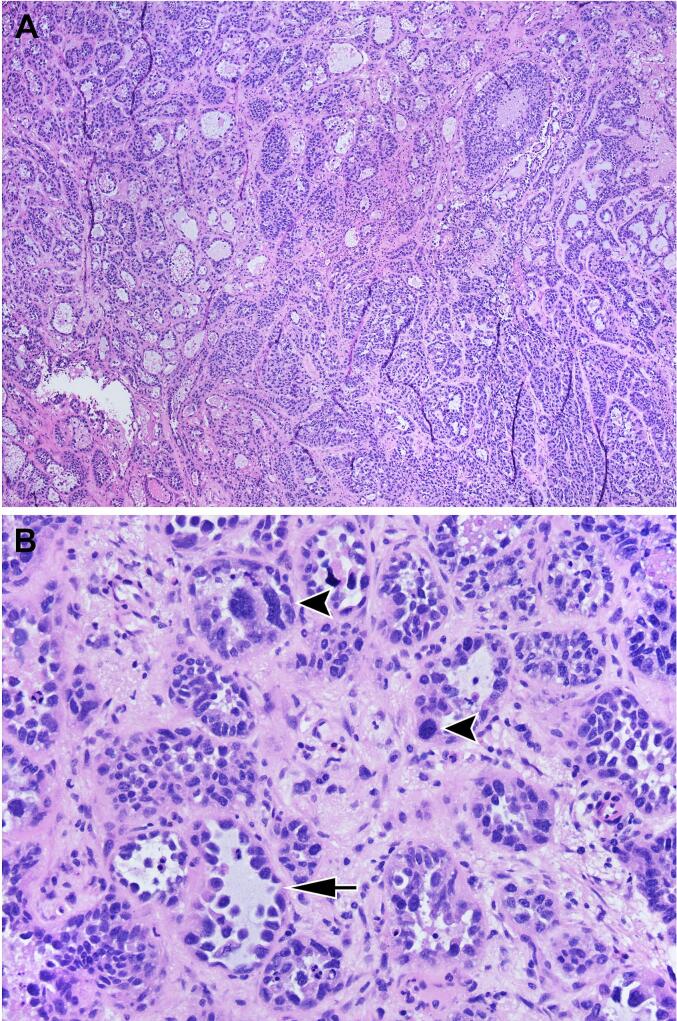
Hematoxylin and eosin (H&E) stains of the sections from the hysterectomy and tumor excision. A. Low power (4x) view of the tumor demonstrating both tubulo-cystic (upper left) and solid (bottom right) growth patterns. B. High power (20x) view demonstrating hyperchromatic cells with hobnail morphology (arrow) and marked atypia (arrowheads).

The incidence of vaginal clear cell adenocarcinoma in the setting of in-utero DES exposure is approximately 1/750 exposed women (Huo et al., 2017). The disease presentation occurs on average between the ages of 15 and 31 however, cases have been observed as late as age 55 (Huo et al., 2017). DES related vaginal clear cell typically originates in the upper 2/3 of the vagina and vaginal lesions are associated 90% of the time with vaginal adenosis (Offman and Longacre, 2012). There are various theories regarding the pathophysiology of adenocarcinoma following DES exposure, but one proposed theory is that adenosis in the upper vagina/cervix is more susceptible to carcinogens and increases cancer risk (Herbst, 2000). The growth pattern of vaginal clear cell adenocarcinoma is most commonly tubulo-cystic composed of small and dilated glands, though papillary, solid, and combinations thereof are seen (Herbst et al., 1971). Focal necrosis, hyaline globules, and psammoma bodies may also be present. The cells are typically round to irregular and hyperchromatic with distinct nucleoli, eosinophilic to clear cytoplasm, scattered marked atypia, and a low mitotic rate of <10 per 10 high-powered fields. They often show a “hobnail” morphology wherein the nuclei protrude into lumens without appreciable cytoplasm (Fig. 1) (Herbst and Scully, 1970). Classically this lesion will have positive immunohistochemical staining with Napsin A and HNF-1β (Herghelegiu et al., 2018).

Approximately 70% of patients are stage I at the time of diagnosis. For early-stage vaginal clear cell, surgical treatment may include radical hysterectomy, upper vaginectomy and lymphadenectomy. In some cases, a less surgically aggressive approach may be preferred using chemotherapy and radiation (Offman and Longacre, 2012). Due to the rarity of primary vaginal carcinoma, the treatment practices are extrapolated from cervical or vulvar data. When vaginal carcinoma is metastatic, progressive, or recurrent out of the pelvis, consideration for a platinum-based chemotherapy doublet typically with paclitaxel had been the mainstay. Since 2014, and the publication of GOG 240, the addition of VEGF inhibitors has become standard of care in appropriately selected patients due to the documented overall survival benefit (Tewari et al., 2014). More recently, the addition of the immune checkpoint inhibitor Pembrolizumab has shown benefit in patients with programmed death ligand (PD-L1) positive cancers that are frequently seen in the cervix, vulva, and vagina (Colombo et al., 2021, Shapira-Frommer et al., 2022). Recurrent tumors that are isolated in the pelvis may be candidates for radiation alone or surgical exenteration procedures (Jhingran, 2022).

## 2 Case

**Fig. 2:**
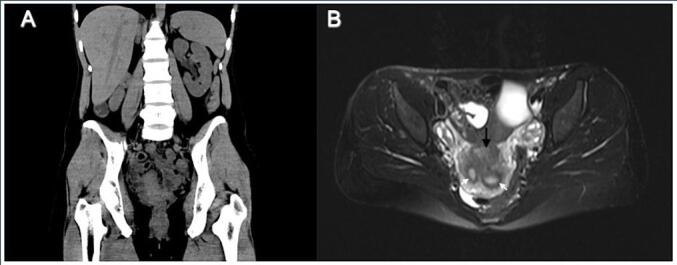
Imaging findings demonstrating anomalous anatomy. A. Coronal CT demonstrating evidence of absent right kidneyB. Axial MRI demonstrating vaginal mass (black arrow) and bicornuate uterus (white arrow noting each uterine horn).

We present the case of a patient with likely DES associated recurrent vaginal clear cell carcinoma who had a complete and durable response to Pembrolizumab. Patient is a G3P2012 who presented at age 38 to care with post-coital bleeding. Past medical history was only notable for Hashimoto’s thyroiditis. Physical exam revealed complete obliteration of the upper vagina and cervix from visible tumor. Biopsy of the tumor returned as vaginal clear cell carcinoma and was ultimately diagnosed with stage II disease. She was unaware if her mother had been exposed to DES or other synthetic estrogens. On imaging, a bicornuate uterus and congenital absence of her right kidney was noted (Fig. 2). Due to the extent of sub-urethral disease, it was felt that she was not a primary surgical candidate so underwent primary chemoradiation with whole pelvic radiation (RT) and weekly cisplatin. She had no evidence of disease (NED) following treatment and entered surveillance.

She had an initial 30-month disease free interval when she presented with pelvic pain. On pelvic ultrasound and MRI there was a non-specific heterogeneous hypo-enhancement involving the cervix and posterior vaginal wall concerning for recurrence. PET/CT at the time showed no evidence of metastatic disease. Recurrence was confirmed with biopsy. As this was a central recurrence it was recommended that she have an anterior exenteration procedure. After a discussion of risks and benefits with the patient decision was made to attempt a bladder sparing surgical approach understanding the risk of further adjuvant treatment if unable to achieve adequate margins. She underwent an exploratory laparotomy, radical hysterectomy due to fibrosis, bilateral salpingo-oophorectomy, and upper vaginectomy. On final pathology, disease was limited to the cervix and vagina, but she had a microscopic positive vaginal margin. She was presented at a multidisciplinary conference with the decision to give stereotactic body radiation therapy (SBRT) to the vaginal margin followed by chemotherapy that included carboplatin, paclitaxel, and bevacizumab once she had healed from surgery. At the end of treatment, she was NED and was returned to surveillance.

During her time in surveillance, she struggled with chronic pelvic pain secondary to vaginal cuff necrosis in which hyperbaric oxygen treatments were used. After 15 months of surveillance, she had vaginal bleeding and was diagnosed with her second recurrence. Immunohistochemical analysis of the tumor revealed PDL-1 + status, with a combined positive score (CPS) of 5. From 5/28/2020 – 4/12/22, she received 19 cycles of Pembrolizumab. Her dosing regimen was Pembrolizumab 200 mg IV every 21 days for the first five cycles, followed by 400 mg every IV every 42 days for the following 14 cycles. Given her history of hypothyroidism, and pembrolizumab’s known inflammatory toxicities, her thyroid function was monitored with each cycle without evidence of superimposed thyroiditis. She did not experience any other direct drug related toxicities. With this regimen, she had a complete response and continues to be NED at 7 months following discontinuation of Pembrolizumab.

## 3 Discussion

This case is of a young patient with recurrent vaginal clear cell carcinoma who showed a complete and durable response to pembrolizumab. While it is unclear if this case represents vaginal clear cell carcinoma because of in-utero DES exposure there are many features that are in line with exposure. Features such as her rare diagnosis of vaginal clear cell carcinoma, younger age of 38 at diagnosis, and Mullerian anomalies such as a bicornuate uterus and absent unilateral kidney are all consistent with DES exposure (Fig. 2). The patient did express that her mother was in the military during her pregnancy and was possibly exposed to DES, though is not completely certain. However, the timeline is not completely consistent as DES was taken off the market a few years prior to her birth.

There has been a growing interest in immunotherapy in the field of oncology. To date, there are no documented cases using pembrolizumab as adjuvant treatment for active or recurrent vaginal clear cell adenocarcinoma. Pembrolizumab is an anti-programmed death 1 (PD-1) monoclonal antibody that blocks the PD-1/PD-L1 pathway. This pathway is an escape mechanism that malignant cells use to evade immune surveillance thereby augmenting T-cell mediated anti-tumor activity (Colombo et al., 2021). Tumors that have higher expression of the program death ligand (PD-L1) as defined by the combined positive score are more likely to respond to Pembrolizumab (Colombo et al., 2021). Immune checkpoint inhibitors such as Pembrolizumab have demonstrated impressive, durable responses even among patients who have undergone multiple lines of prior systemic therapy in multiple types of cancers (Colombo et al., 2021, Shapira-Frommer et al., 2022, Cortes et al., 2020). In the phase III randomized control trial KEYNOTE 826 in patients with persistent, recurrent, or metastatic cervical cancer including patients who had adenocarcinoma showed improved progression and overall survival by the addition Pembrolizumab to chemotherapy plus or minus bevacizumab (Colombo et al., 2021). In addition to the efficacy of Pembrolizumab another advantage is the tolerability of the drug. Common side effects tend to be mild such as fatigue and nausea. However, in rare circumstances, immune-mediated drug reactions can occur in organs such as the lung, liver, skin, and thyroid (Flynn and Gerriets, 2023).

Vaginal cancer represents 1–2% of all gynecological cancers. The incidence rates of vaginal clear cell carcinoma have become so rare since the discontinuation of DES that they approach less than 0.1% for patients born on the last year that DES was available in the United States (Huo et al., 2017). As expected, it is difficult to perform a randomized controlled trial assessing the effects of pembrolizumab on clear cell vaginal cancer. However, our case provides meaningful insight for future research and treatment considerations using Pembrolizumab.

## CRediT authorship contribution statement

**Hector S. Porragas-Paseiro:** Writing – original draft, Visualization, Writing – review & editing. **Saketh Guntupalli:** Conceptualization, Writing – review & editing. **Jessie Xiong:** Writing – review & editing, Visualization. **Ashley Greenwood:** Conceptualization, Writing – original draft, Supervision.

## Declaration of Competing Interest

The authors declare that they have no known competing financial interests or personal relationships that could have appeared to influence the work reported in this paper.
